# Induced membrane technique using bone cement with or without cefazolin in chicken segmental radius defect

**DOI:** 10.3389/fvets.2023.1027951

**Published:** 2023-03-07

**Authors:** Luiz D. Campeiro Junior, Sheila Canevese Rahal, Marcos A. Souza, Alini Osowski, José I. S. Silva Júnior

**Affiliations:** ^1^Department of Veterinary Surgery and Animal Reproduction, School of Veterinary Medicine and Animal Science, São Paulo State University (UNESP), Botucatu, Brazil; ^2^Department of Veterinary Pathology, Mato Grosso School of Veterinary Medicine, Cuiabá, Brazil

**Keywords:** spacer, membrane, antibiotic, polymethyl methacrylate, histology, radiography

## Abstract

The utilization of antibiotic-loaded cement spacer in the induced membrane development has been a debate topic in human medicine. To the best of the author's knowledge, these combinations have not yet been evaluated in birds. Therefore, this study assessed induced membrane formation using radiography and histology, in a segmental defect of a chicken radius, with or without the addition of cefazolin. Thirty 18-month-old healthy chickens were divided into two equal groups: G1—bone defect filled with bone cement; G2—bone defect filled with cefazolin powder-loaded bone cement. Radiographic examinations of the left forearm were taken immediately after surgery and at 7, 15, and 21 postoperative days. For the collection of the induced membranes, five chickens in each group were euthanized at 7, 15, and 21 days after surgery. Radiographically, the bone cement was identified as a radiopaque structure occupying the bone defect in both groups. Mild new bone formation in at least one of the fractured extremities of the bone defect was seen only 21 days after surgery in most chickens. Histologically, there was no difference in the mean thickness of the induced membrane between groups at all time points. Multifocal multinucleated cells differed between groups at 7 (G1 > G2) and 21 (G2 > G1) days after surgery. Mononuclear inflammatory infiltrate differed between groups only on day 21 (G1 > G2). Fibrous tissue proliferation did not differ between groups at all evaluation times. Blood vessel density differed only at 21 days postoperatively (G2 < G1). Multifocal areas of cartilage differed between groups at all time points (G1 > G2). In conclusion, cefazolin mixed with bone cement did not affect thickness of the induced membrane, but did result in a negative effect on some histological aspects, such as fewer vessels, less multifocal areas of cartilage, and persistence of inflammation.

## Introduction

Strategies for the management of bone damage and infections, which cause large segmental bone defects, have been constantly improved in human patients (1, 2). Some treatment strategies have also been applied to veterinary patients, including birds, with reports of distracting osteogenic, bone transport, or use of the demineralized bone matrix (3–5). The choice of treatment in avian should be guided by factors such as the complexity of the surgical procedure and learning curve, bone involved, method of stabilization, cost, and prognosis for return to function, among others.

Masquelet's induced membrane technique has been introduced in humans as a less complex option to treat large bone defects compared to bone transport and vascularized bone grafts (6–8). The procedure is carried out in two steps. In the first step, debridement of the compromised area is performed, and polymethyl methacrylate-based bone cement is placed in the bone defect to act as a spacer and membrane inducer (6, 9–11). In the second step, the spacer is removed after 6 to 8 weeks and the defect is filled with an autologous graft (7, 8, 10). The induced membrane is thought to assist with revascularization, prevent graft resorption and improve corticalization (6, 9, 11). In birds, the characteristics of the induced membrane were assessed in chickens at 15, 21, and 30 days of permanence of bone cement (12).

The development and characteristics of the induced membrane may be influenced by additives, such as barium sulfate (7) and antibiotics (13). However, there are controversies regarding the validity of the use or not of antibiotic*-*loaded bone cement in the development of an induced membrane in humans (6, 7, 14). To the best of the author's knowledge, the use of antibiotic-loaded bone cement in the induced membrane technique has not been evaluated in birds. The bones of birds have a number of differences to those of mammals, including higher calcium content that contributes thin outer shell and brittle nature, a fast bone healing rate, and the presence of pneumatic bones (15, 16), and this may influence the results.

Thus, this study aimed to evaluate through radiographic and histological examinations the induced membrane technique using bone cement, with or without cefazolin, in a segmental defect in the left radius of chickens.

## Materials and methods

### Subjects

Thirty healthy female chickens (*Gallus domesticus*) purchased from commercial breeding, with a mean age of 18 months and weighing from 1 to 1.8 kg (mean ± SD: 1.4 ± 0.3 kg) were used. The chickens were kept in an area (4.0 m wide x 12.0 m long x 3.0 m high) with natural ventilation, and received a commercial diet and water *ad libitum*. The chickens were randomly divided into two equal groups: Group 1 – bone defect filled with bone cement; Group 2 –bone defect filled with cefazolin powder-loaded bone cement.

### Anesthesia and surgical procedure

After 6 h of fasting, each chicken received tramadol hydrochloride (8 mg/kg), midazolam (1 mg/kg) and ketamine hydrochloride (5 mg/kg), intramuscularly in the pectoral muscle. General anesthesia was induced by a face mask and maintained with 5% isoflurane administered through a non-cuffed endotracheal tube (2.5% isoflurane in a 1.5-L flow of oxygen). Blood pressure, heart rate, respiratory rate, end-tidal CO2, and pulse oximetry were monitored.

After plucking the feathers from the left antebrachium, the chicken was placed in dorsal recumbency on a heating pad. The skin was aseptically prepared, and the surgical procedure was conducted as described in chickens (12). Briefly, a longitudinal skin incision was made on the ventral aspect of the radius, and the extensor metacarpi and the pronator muscles were identified and retracted to expose the bone. An oscillating saw was used to create a 1.5 cm segmental defect in the radius diaphysis. In Group 1, the bone defect was filled with bone cement (C-VET; CIMTECH, Rio Claro, São Paulo, Brazil) in a cylindrical shape still in the dough phase. The muscles and fascia were apposed with a simple continuous pattern using 5-0 nylon. A simple interrupted pattern was used in closing the skin incision with a nylon monofilament 3–0 suture. In Group 2, the bone defect was filled with cefazolin powder-loaded bone cement (0.104 g of cefazolin per 0.54 g of bone cement) (Cefazolina sódica; ABL, Cosmópolis, SP, Brazil) in a cylindrical shape still in the dough phase. For this purpose, after cement powder and cefazolin powder were mixed in a sterile mixing bowl, the liquid monomer was added. The combination was hand mixed using a spatula. The liquid component of the orthopedic cement contained Monomethyl Methacrylate, NN Dimethyl-p–toluidine, and Hydroquinone, while the powder contained Polymethyl Methacrylate, Barium Sulfate, and Benzoit Peroxide. According to the manufacturer instructions, the bone cement was sterilized with ethylene oxide.

A single dose of cefazolin (15 mg/kg IM) was administered to all chickens before the surgical procedure. Meloxicam was administered (0.5 mg/kg q 24 h IM) for 6 days. The wounds were cleaned daily with 2% chlorhexidine and saline solution and bandaged. The skin sutures were removed 7 to 10 days after surgery.

### Radiographic examination and histological analysis

Radiographic examinations of the left forearm were taken in mediolateral and craniocaudal views immediately after surgery and at 7, 15, and 21 days postoperatively. The positioning of the cement spacer into the bone defect (correctly positioned; slightly, mild, or moderate displacement) and bone proliferation (present or absence) were evaluated.

For histological analysis, five chickens in each group were euthanized at 7, 15, and 21 days after surgery. Therefore, after sedation with ketamine hydrochloride (30 mg/kg IM), xylazine hydrochloride (4 mg/kg), and tramadol (8 mg/kg IM), the chickens were deeply anesthetized with isoflurane, followed by intravenous potassium chloride injection until cardiac arrest. Then, a longitudinal incision was done in the skin over the original surgical area, and the muscles were carefully removed to expose the induced membrane. The membrane was cut longitudinally with caution, and the bone cement was removed. Adherence between the bone cement and induced membrane was not observed. Then, the total induced membrane was collected and fixed in 10% neutral-buffered formalin. Subsequently, paraffin-embedded membranes were cut into sections 2–3 μm*-*thick and stained with Hematoxylin-Eosin. In addition, the fractured bone ends were also collected, decalcified in 10% EDTA solution, followed by dehydration in alcohol and clarification with xylene. Then, the bone ends were embedded in a paraffin block, cut into 5 μm-thick slices, and stained with Hematoxylin-Eosin.

**Figure 1:**
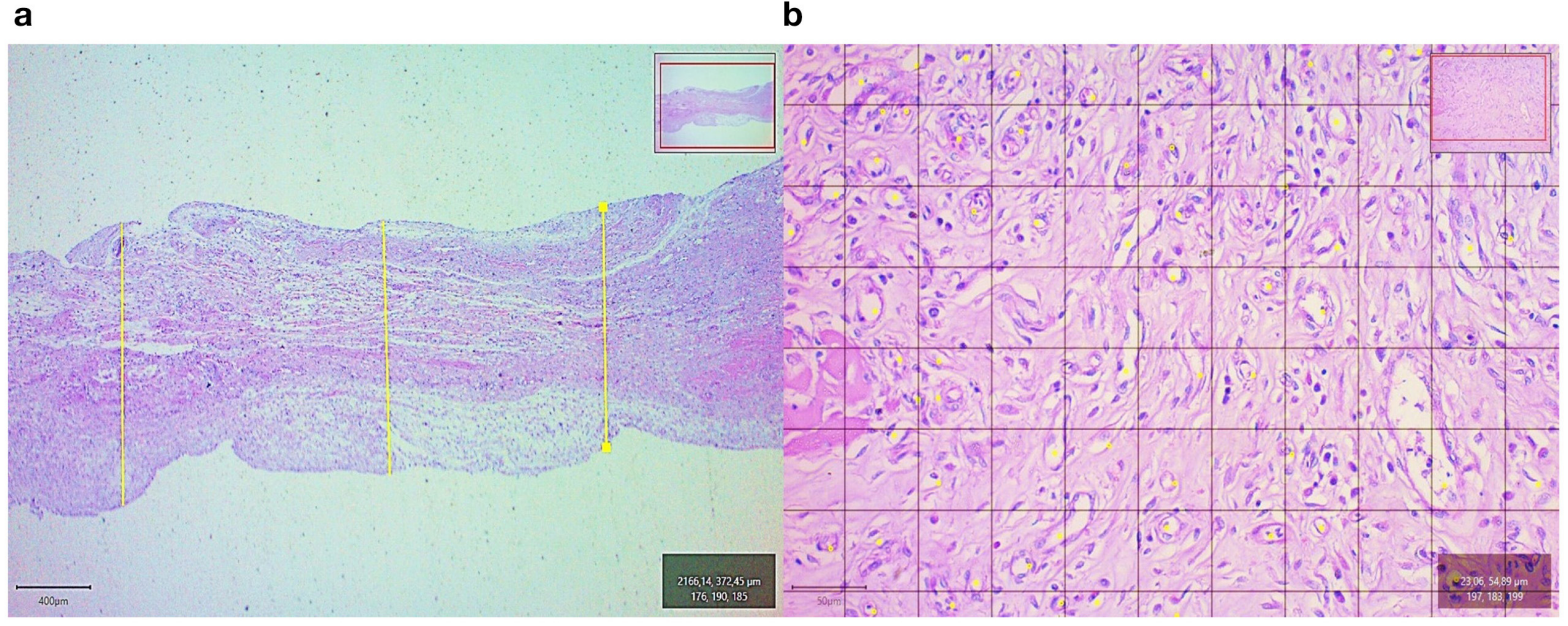
**(a)** Thickness measurement of the induced membrane at three different sites (yellow lines) (HE, 2.5x) and **(b)** blood vessel counting (yellow dots) (HE, 20x) in a chicken from Group 1 (bone cement) on day 21 after surgery, using the QuPath-0.3.2 software.

For the induced membranes, the type and extend of the inflammatory reaction, fibrous tissue proliferation, and presence of multifocal areas of cartilage were assessed qualitatively in a blind manner. For fractured bone ends, the proliferation of bone matrix was also evaluated qualitatively. These microscopic findings were assigned a degree of intensity: absent (-), mild (+), moderate (++), and accentuated (+++). The quantitative assessment of the induced membrane included thickness (in μm) and density of blood vessel. Digital images (1,600 x 1,200 pixels; 32 bits/pixel; RGB) of the histological slides were captured using an optical microscope (Zeiss Axio Scope A1; Germany). The thickness of the induced membrane was measured at 2.5X magnification in three different locations (Figure 1a). A total of nine measurements per membrane/chicken were done at 7, 15, and 21 days after surgery. For assessing blood vessel density, digital images were captured using the 20X objective and superimposed by a grid (50 x 50 μm) (Figure 1b). Manual count of the blood vessels was performed. The inclusion criteria included structures composed from one to three layers and covered by vascular endothelium, with or without red blood cell. All image analysis was performed with the QuPath software (version 0.3.2).

### Statistical analysis

Kolmogorov-Smirnov test was performed to determine if data were normally distributed. For the variables with normal distribution (thickness of the induced membranes, and blood vessel density), a student *t-*test was performed to compare the two groups at each time point (7, 15, and 21 days after surgery), and analysis of variance (ANOVA) was carried out to evaluate inside each group at each time point. For the evaluation of induced membrane and fracture bone end microscopic changes (inflammatory reaction, fibrous tissue proliferation, multifocal areas of cartilage of the induced membrane, and bone matrix in fractured bone ends), the percentage of chickens with each grade was determined, and then analyzed by group and time point. The chi*-*square (χ2) test was used to compare the percentages. Differences were considered statistically significant at i < 0.05. Statistical analyses were done using the software BioStat 5.0 and SigmaStat 3.5.

## Results

### Clinical and radiographic evaluations

There were no complications after surgery. The wounds healed uneventfully.

**Figure 2:**
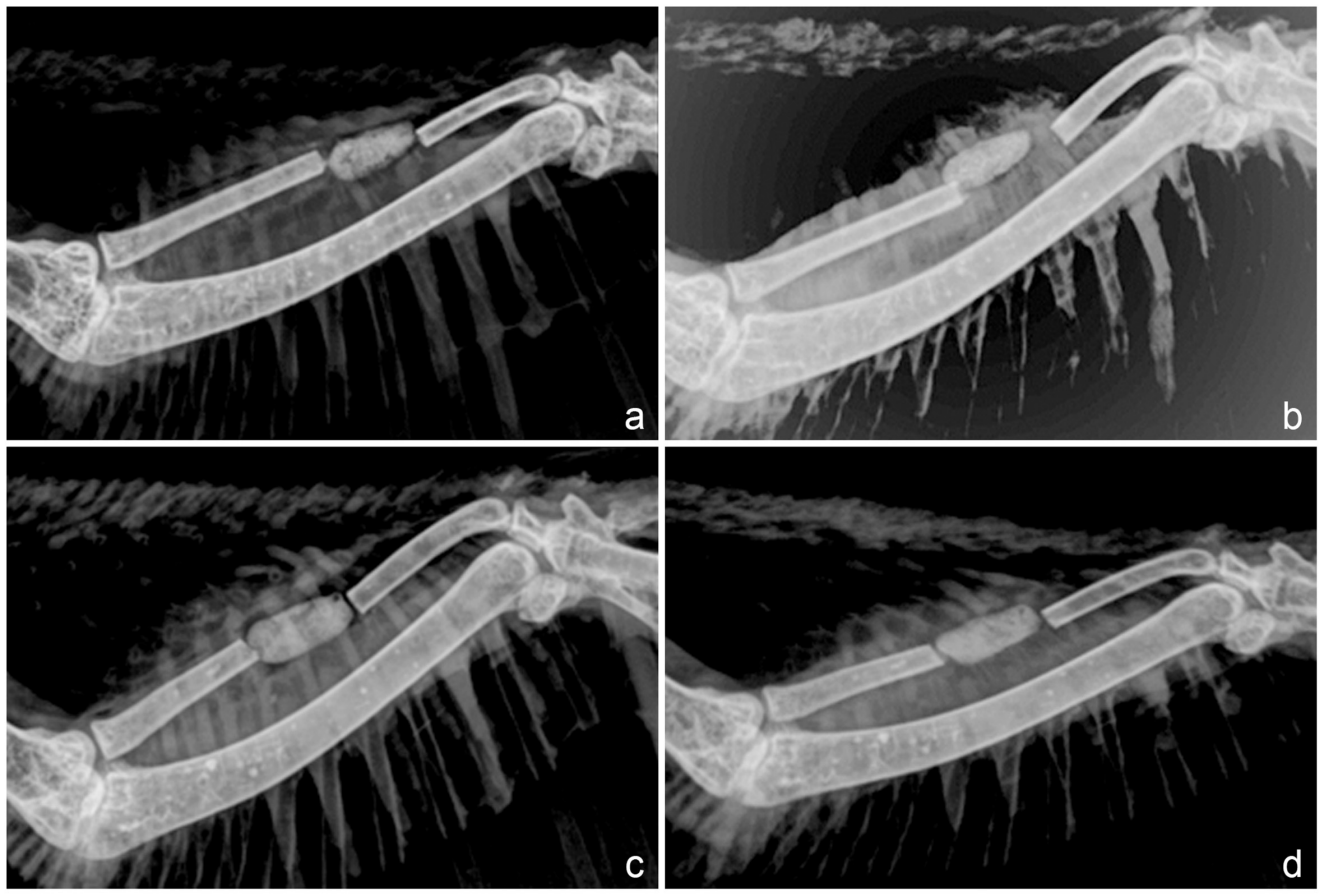
Mediolateral radiographic views of the antebrachium of chickens immediately after surgery **(a, c)** and on day 21 postoperative **(b, d)**. Group 1 **(a, b)** corresponds to the bone defect filled with bone cement, and Group 2 corresponds to the bone defect filled with cefazolin powder-loaded bone cement **(c, d)**. Observe in both groups the bone cement as a radiopaque structure occupying the bone defect. Note the displacement of the bone cement on day 21 postoperative in Group 1 **(b)**.

Radiographically (Figure 2), the bone cement was identified as a radiopaque structure occupying the bone defect in both groups at all time points. Mild to moderate displacements of the bone cement were detected in 57.22% of a total of 30 chickens evaluated. Mild new bone formation in at least one of the fractured extremities of the bone defect was seen only 21 days after surgery in 97% of the chickens.

### Histological evaluation of the induced membrane

**Figure 3:**
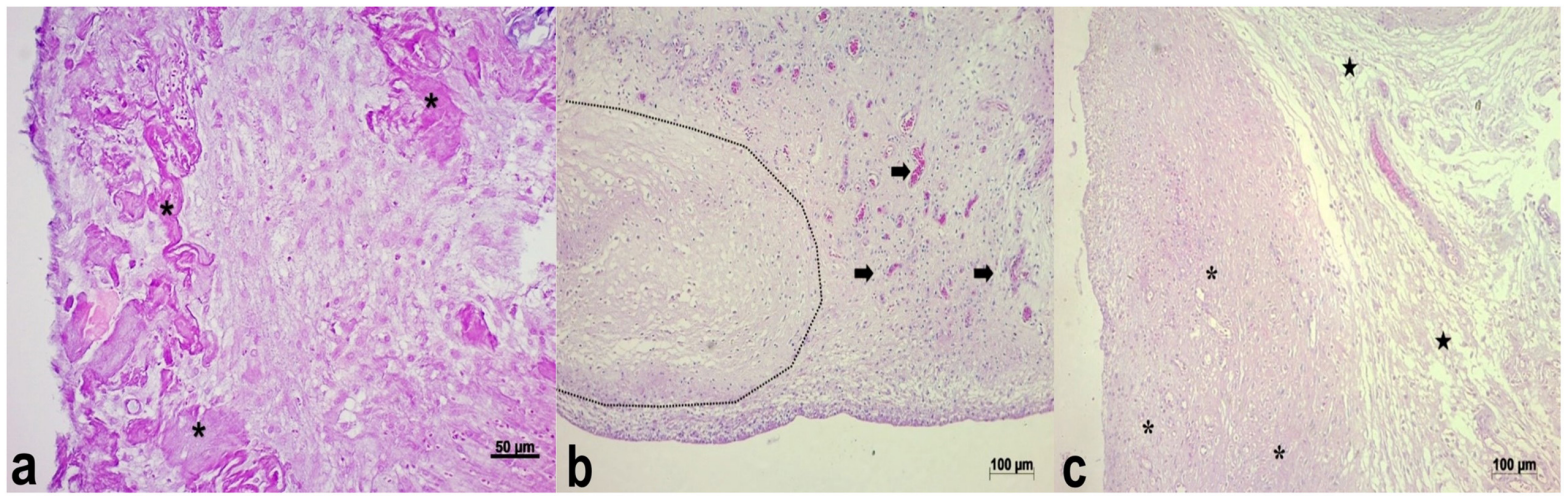
Microscopic appearance of the induced membrane in chickens with bone cement at seven **(a)**, 15 **(b)**, and 21 **(c)** postoperative days. **(a)** Fibrovascular tissue surrounding fragmented and hypereosinophilic necrotic muscle fibers (asterisks). HE, 20x. **(b)** Marked proliferation of fibrous, disorganized and highly vascularized tissue (arrows) with an area of fibrocartilage (dotted line). HE, 10x. **(c)** Moderate, disorganized, and slightly vascularized fibrous tissue proliferation (asterisks), and loose connective tissue (stars) close to skeletal muscle tissue. HE, 5x.

**Figure 4:**
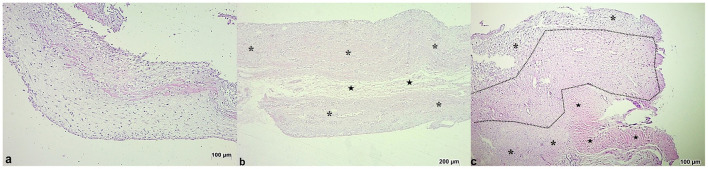
Microscopic appearance of the induced membrane in chickens with cefazolin powder- loaded bone cement at seven **(a)**, 15 **(b)**, and 21 **(c)** postoperative days. **(a)** Fibrous and thin membrane showing low density (loose matrix), and an eosinophilic amorphous proteinaceous material at the center. HE, 10x. **(b)** Dense fibrous tissue (asterisks), narrow band of loose connective tissue (stars). HE, 5x. **(c)** Proliferation of loose connective tissue (asterisks), extensive area of cartilaginous tissue (dotted line), and skeletal muscle tissue (stars). HE, 10x.

In both groups, on day 7 postoperatively, a fibrovascular tissue composed of fusiform cells with clear nuclei (interpreted as fibroblasts), as well as polygonal cells with ample cytoplasm and a clear and slightly rounded nucleus (interpreted as histiocytes) were observed. In addition, there were multiple areas of fragmented eosinophilic material (necrotic muscle fiber) (Figures 3a, 4a). In each group, the induced membrane was not identified in one chicken.

At 15 days postoperatively, fibrous tissue composed of polygonal to fusiform cells was seen in both groups. The tissue was disorganized, loose, and moderately to highly vascularized (Figures 3b, 4b). In Group 1, a focal area of myxoid metaplasia, and multifocal areas of cartilage were also verified (Figure 3b). In Group 2, there was also a mononuclear inflammatory infiltrate composed mainly of histiocytes, in addition to degenerated muscle fibers.

**Figure 5:**
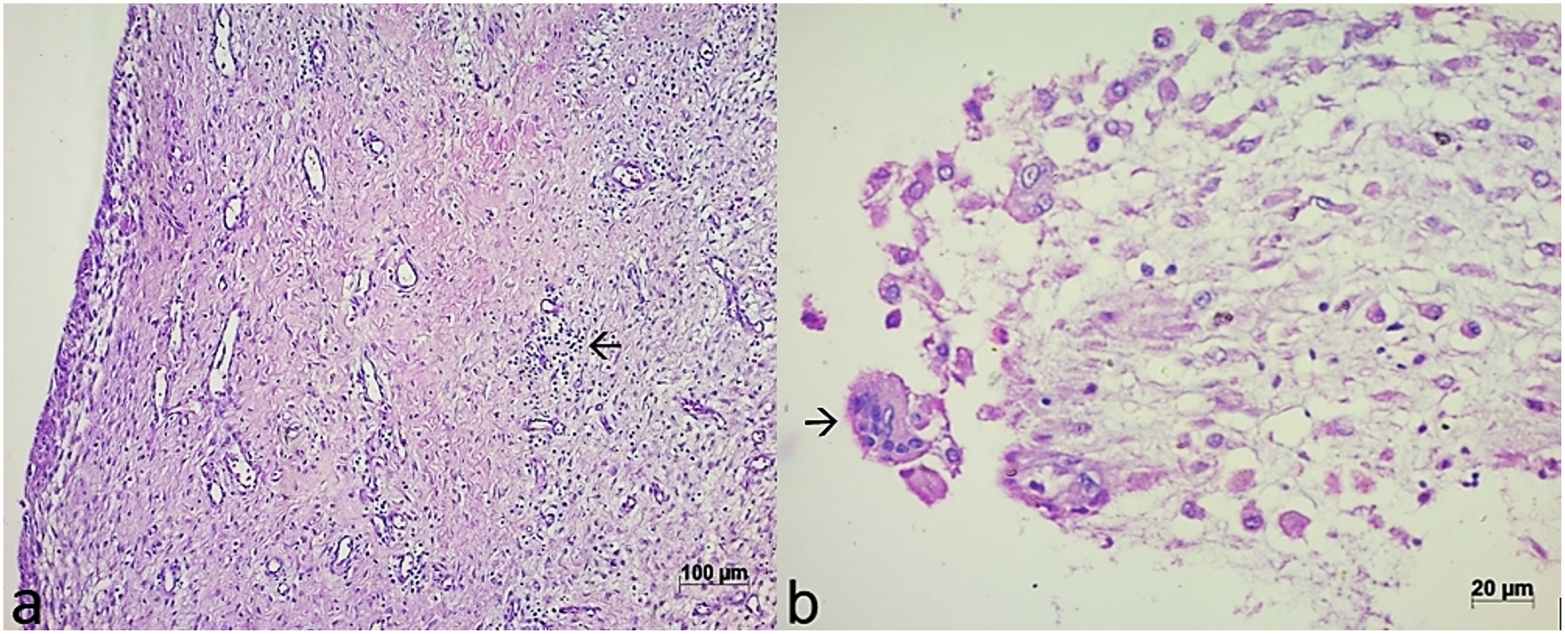
Microscopic appearance of the induced membrane in a chicken with bone cement on day 21 postoperative. **(a)** Note the dense proliferation of fibrous connective tissue, highly vascularized and with mononuclear inflammatory infiltrate (arrow). HE, 20x. **(b)** Microscopic appearance of the induced membrane in a chicken with cefazolin powder-loaded bone cement on day 21 postoperative. Observe inflammatory infiltrate composed mainly of mononuclear inflammatory cells and multinucleated cells (arrow). HE, 40x.

At 21 days after surgery, a fibrous tissue composed of polygonal to spindle-shaped cells, disorganized and highly vascularized, was identified in both groups (Figures 3c, 4c). However, two very distinct layers (dense and loose) were observed in only one chicken in each group. In both groups there was also a mononuclear inflammatory infiltrate composed mainly of histiocytes (Figure 5).

The multinucleated cells were only qualitatively evaluated (present or absent). Statistical analysis of the histological findings revealed that the presence of multifocal multinucleated cells differed between groups at 7 (G1 > G2) and 21 (G2 > G1) days after surgery (*p* < 0.001). There were differences within each group (*p* < 0.001), with the lowest percentage of membranes with multifocal multinucleated cells in Group 1 on day 21, and Group 2 on day 7 postoperative. The mononuclear inflammatory infiltrate showed no difference within each group (*p* = 1.00), but differed (*p* = 0.046) between groups only on day 21 (G1 > G2). The percentage of membranes with fibrous tissue proliferation did not differ between groups (*p* = 1.00) or within the same group at all evaluation time points (*p* = 0.239). The percentage of membranes with multifocal areas of cartilage differed between groups (*p* < 0.001) at all time points (G1 > G2). Within each group, Group 2 did not present multifocal areas of cartilage at 15 and 21 days after surgery, while Group 1 had higher multifocal areas of cartilage at 7 days compared to 15 and 21 days after surgery (*p* < 0.001).

In both groups, the mean of the induced membrane thickness was lower at seven post-operative days (*p* < 0.05) compared to 15 and 21 postoperative days, which did not differ from each other. There was no difference in thickness between groups (*p* > 0.05). In Group 1, blood vessel density in induced membranes was lower at 7 postoperative days (*p* = 0.016) compared to 15 and 21 postoperative days, which did not differ from each other. In Group 2, there was no difference among time points (*p* = 0.296). Between groups, there was lower blood vessel density in Group 2 compared to Group 1 only at 21 days postoperatively (*p* = 0.041).

Table 1 shows the statistical comparison of histological findings between groups.

**Table 1:** Statistical comparison^*^ of histological findings of the induced membranes in Group 1 (bone defect filled with bone cement) and Group 2 (bone defect filled with cefazolin powder-loaded bone cement) at seven, 15, and 21 postoperative days.

**Variables**	**7 days**	**15 days**	**21 days**
Multifocal multinucleated cells	G1 > G2 (*p* < 0.001)	G1 = G2 (*p* = 1.00 )	G2 > G1 (*p* < 0.001)
Mononuclear inflammatory infiltrate	G1 = G2 (*p* = 1.00)	G1 = G2 (*p* = 1.00)	G1 > G2 (*p* = 0.046)
Percentage of membranes with fibrous tissue proliferation	G1 = G2 (*p* = 1.00)	G1 = G2 (*p* = 1.00)	G1 = G2 (*p* = 1.00)
Percentage of membranes with multifocal areas of cartilage	G1 > G2 (*p* < 0.001)	G1 > G2 (*p* < 0.001)	G1 > G2 (*p* < 0.001)
Induced membrane thickness	G1 = G2 (*p* = 0.604)	G1 = G2 (*p* = 0.725 )	G1 = G2 (*p* = 0.094)
Blood vessel density in induced membranes	G1 = G2 (*p* = 0.772 )	G1 = G2 (*p* = 0.419)	G2 < G1 (*p* = 0.041)

^*^Differences were considered statistically significant at *p* < 0.05.

### Histological evaluation of fractured bone ends

**Figure 6:**
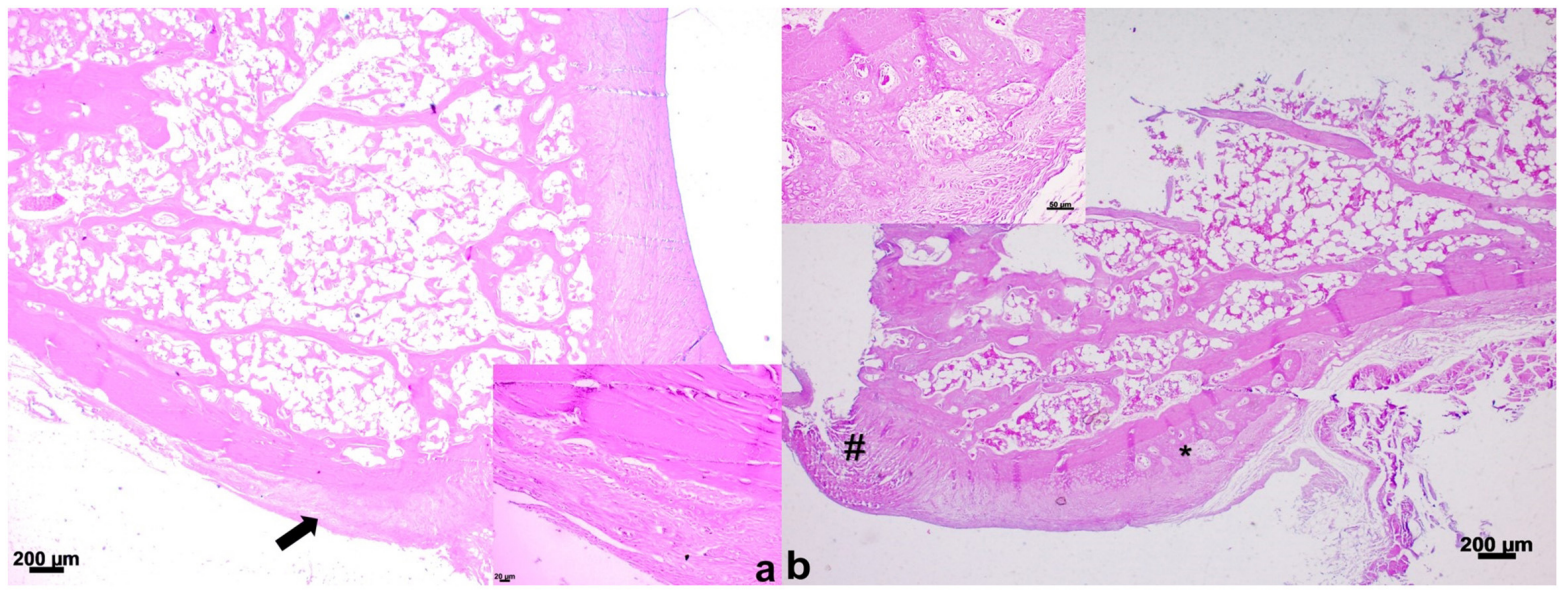
**(a)** Proximal fractured end of the radius in a chicken of Group 2 on day 21 after surgery. Observe slight thickening (arrow) of the periosteum (HE, 10x); Insertion—Note a slight proliferation of bone tissue under the periosteum (HE, 20x). **(b)** Distal fractured end of the radius in a chicken of Group 2 on day 21 after surgery. Observe disorganized and fibrillary material (hashtag), and periosteal new bone (asterisk) (HE, 10x). Insertion—Note a moderate proliferation of bone and vascular tissue under the periosteum (HE, 20x).

Proliferation of bone matrix occurred at the fractured bone ends, mainly from the periosteum. This bone matrix was considered disorganized and immature at 15 and 21 days. There was a mild to moderate endosteal reaction adjacent to the fracture site at 21 days. The percentage of animals with bone matrix of the proximal fractured bone ends differed within each group (*p* < 0.001) and between groups at 15 days (G2 > G1) and 21 days (G1 > G2) after surgery (*p* = 0.0098) (Figure 6a). On day 7 the bone matrix was not observed in both groups. Significant differences were also observed in the distal fractured bone ends between groups at 7 (G1 > G2) (*p* < 0.0001) and 21 days after surgery (G2 > G1) (*p* = 0.0016). Within each group, only Group 2 showed higher bone matrix at 21 days compared to 7 and 15 days after surgery (*p* < 0.001) (Figure 6b).

## Discussion

The present study evaluated the development of the induced membrane using bone cement with or without cefazolin, with mainly histological differences found within the groups. The utilization of antibiotic-loaded cement spacer, as used in the current study, has been a topic of debate in human patients and cannot be considered a complement to inadequate surgical debridement (7). The method has already been used in clinical cases of infected non-union in humans with good results (6, 14). However, Masquelet does not recommend it due to the risk of antibiotic resistance; some antibiotics may influence the structural aspects of induced membrane; absence of infection recurrence using cement without antibiotics is favorable evolution indicative (7).

In the current study, the antibiotic used was cefazolin, a first-generation cephalosporin (15). Many antibiotics can be heat labile or promote a deleterious effect on cement (17) that needs to be considered when selecting an antibiotic to be incorporated into polymethyl methacrylate. Studies of cefazolin elution from polymethyl methacrylate as beads or blocks have shown that after the initial release, the concentration decreases while maintaining the minimum inhibitory concentration (18–20). The total dose of cefazolin contained in the cement was 100 mg in the present study. The systemic dosage of cefazolin has been reported as 25–75 mg/kg q 8–12 h for most avian species (15). Since pharmacokinetic data regarding preparations of antibiotic-impregnated bone cement in veterinary patients is considered limited, consideration should be given to simultaneous parenteral dosing (21).

No postoperative complications were observed in either group. In turn, an induced membrane study without adding antibiotics to the bone cement observed signs of inflammation with edema and serous discharge in two chickens (12). In both groups, mild new bone formation in at least one of the fractured extremities of the bone defect was seen on radiographs only at 21 days postoperatively. These findings are in accordance with the histological analysis since bone matrix did not show a uniform pattern between fractured bone ends and groups, with the highest values in bone matrix at 15 and 21 days after surgery.

The bone cement used in the current study had barium sulfate in the powder component. Barium sulfate and zirconium dioxide are the most common radiopacifiers of bone cement (22, 23) and allow bone cement visibility in radiographs (24) as verified in both groups. The displacement of the bone cement observed on the radiographs was also detected in studies with chickens (12) and rabbits (25), and may occur when paired bones are used to maintain stability without a bone fixation method. Thus, a bone plate and screws should be applied to avoid cement displacement.

Histologically, there were no differences between groups regarding the mean thickness of the induced membranes, suggesting that the antibiotic did not interfere with this parameter. In turn, a study in rats observed that cement supplemented with gentamycin and clindamycin caused decreased membrane thickness compared to other groups (13), showing the importance of antibiotic choice. In both groups the mean of the induced membrane thickness was lower at seven postoperative compared to 15 and 21 post-operative days. Conversely, in a study with bone cement without antibiotics in chickens at 1 year of age, the induced membranes at 15 days were considered less thick than at 21 and 30 days postoperatively using macroscopic evaluation (12). The age difference between the studies must be regarded since the chickens used in the current study had mean age of 18 months.

The presence of multifocal multinucleated cells differed between groups at two-time points, with the lowest value in Group 1 on day 21, suggesting a persistence of the inflammatory process in Group 2, probably related to the use of antibiotic. Differences were observed regarding the mononuclear inflammatory infiltrate between groups only on day 21 (G1 > G2). Its presence is justified since the induced membrane is a foreign-body membrane developed in response to the immune system to bone cement (8). The less numerous mononuclear inflammatory in Group 2 may be associated with the persistence of the inflammation, as mentioned above. In addition, the portion of the induced membrane in contact with the cement spacer is described as like-synovium epithelium, and the outer fibrous layer is composed of fibroblasts, myofibroblasts, and collagen bundles with highly aligned fibers (1, 8–10, 26). In the current study, the proliferation of fibrous tissue did not differ between groups or within the same group at all evaluation times, indicating that the antibiotic did not change this variable.

Another structural feature of the membrane is being richly vascularized (1, 7, 26). The blood vessel density in induced membranes was lower on day 7 compared to days 15 and 21 postoperatively only in Group 1. In addition, Group 2 showed lower blood vessel density compared to Group 1 on day 21 after surgery, suggesting a negative effect of the antibiotic on vascular neoformation. The well-vascularized membrane is necessary for the second stage of the Masquelet technique to protect and vascularize the bone graft (8, 9). In addition, the membrane secretes growth factors that enhance the healing of the bone graft (7, 10).

The histological differentiation of the induced membrane layers was identified in one chicken in each group only on day 21 postoperatively. However, multifocal areas of cartilage were observed at all time points in Group 1 and only on day 7 postoperatively in Group 2. These findings differed from a study with chickens in which the induced membrane layers were identified on day 15 after surgery, being this moment considered the most suitable for the second stage of bone reconstruction since cartilage and bone metaplastic areas were detected in induced membranes on day 21 day after surgery (12). The difference in chicken ages may have contributed to the results and must be considered to choose the time of the second stage of the technique.

One of the limitations of the present study is the lack of immunohistochemical analysis, which could provide additional information about induced membranes, including those related to cell proliferation and the differentiation between mature and immature blood vessels. Therefore, further studies are required to include this analysis.

## Conclusion

The cefazolin mixed with bone cement did not affect the thickness of the induced membrane but presented a negative effect on some histological aspects, such as fewer vessels, less multifocal areas of cartilage, and persistence of inflammation.

## Data availability statement

The original contributions presented in the study are included in the article/supplementary material, further inquiries can be directed to the corresponding author.

## Ethics statement

The animal study was reviewed and approved by the Ethics Committee for the Use of Animals from School of Veterinary Medicine and Animal Science, Unesp Campus Botucatu, SP-Brazil (CEUA—No. 0108/2020).

## Author contributions

LC and SR contributed to conception and design of the study. AO was responsible for anesthesia. MS performed the histological analysis and prepared histological figures. LC, SR, and JS wrote the original draft of this manuscript. All authors contributed to manuscript revision, read, and approved the submitted version.
